# Evaluation of combination therapy duration for endocarditis secondary to Enterobacterales

**DOI:** 10.1128/aac.00357-26

**Published:** 2026-05-28

**Authors:** Sunish Shah, Jake Smith, Brandon J. Smith, Ryan K. Shields

**Affiliations:** 1 Antibiotic Management Program, University of Pittsburgh Medical Center 25817, Pittsburgh, Pennsylvania, USA; 2 Department of Pharmacy, University of Pittsburgh Medical Center 25817, Pittsburgh, Pennsylvania, USA; 3 Division of Infectious Diseases, Department of Medicine, University of Pittsburgh 6614, Pittsburgh, Pennsylvania, USA; Columbia University Irving Medical Center, New York, New York, USA

**Keywords:** combination, duration, enterobacterales, endocarditis

## Abstract

Combination antimicrobial treatment for endocarditis secondary to Enterobacterales may be associated with clinical benefits, but this benefit comes with an increased risk of adverse drug reactions (ADRs). We hypothesized that short-course combination therapy would optimize clinical benefits while minimizing ADRs. This was a retrospective, multicenter study of adult patients with definitive endocarditis secondary to Enterobacterales. Short-course combination treatment was defined as receipt of combination therapy for ≤14 days. Seventy-three patients met the inclusion criteria. *Serratia marcescens* was the most common pathogen and was recovered in 62% (45/73) of patients. Patients who received short-course combination treatment were more likely to be admitted prior to 2016 (44% [12/27] vs 17% [8/46], *P* = 0.012) and to receive an aminoglycoside in combination regimens (63% [17/27] vs 28% [13/46], *P* = 0.013). Patients who received short-course combination treatment had a shorter post-infection length of stay (18 [13–25] days vs 29 [20–42], *P* = 0.021), but no significant difference in the rate of discontinuation of antimicrobial therapy due to an ADR (26% [7/27] vs 24% [11/46], *P* = 0.847). In the multivariate logistic regression model, short-course combination treatment was not associated with a higher risk of 90-day mortality (OR = 2.89; 95% CI: 0.63–13.35; *P* = 0.175). This is the first study to compare short- and long-duration combination therapy in patients with Enterobacterales endocarditis. Overall, shorter courses of combination therapy were commonly prescribed, which supports future studies that are designed to identify the optimal duration of combination therapy for patients with Enterobacterales endocarditis.

## INTRODUCTION

The decision to administer combination antimicrobial regimens for non-HACEK gram-negative endocarditis has remained controversial. However, consistency has been observed when pathogens have been studied in silo. For instance, in every study exclusively evaluating endocarditis secondary to *Serratia* spp., combination therapy has been shown to be of clinical benefit (1–4). Conversely, no study that exclusively evaluates patients with *P. aeruginosa* endocarditis has shown combination therapy to be associated with improved outcomes compared to monotherapy (5, 6). More recently, in a propensity matched study of patients with endocarditis secondary to non-*Serratia* Enterobacterales, a trend toward decreased mortality was observed in those who received combination therapy, and treatment emergent resistance was only observed among those who received monotherapy (7). Although combination antimicrobial treatment for endocarditis secondary to *Serratia* and other Enterobacterales has been associated with clinical benefits, it has also been associated with an increase in adverse drug reactions (ADRs) (1, 5, 7).

It may be postulated that shorter courses of combination therapy prior to narrowing to monotherapy could mitigate ADRs. However, current guidelines recommend 6 weeks of combination therapy for endocarditis secondary to Enterobacterales, and to date, no studies have evaluated the duration of combination therapy for gram-negative endocarditis (8). Therefore, the purpose of this study was to compare clinical outcomes of patients who received short-course combination treatment and long-course combination treatment among patients with Enterobacterales endocarditis.

## MATERIALS AND METHODS

This was a retrospective, multicenter study of adult patients with definitive endocarditis secondary to Enterobacterales between January 2000 and November of 2025 across 11 hospitals. The study was approved by the University of Pittsburgh Institutional Review Board (STUDY21110056) with a waiver of written informed consent. Patients were excluded if they received <48 hours of combination therapy, exclusively received monotherapy, or experienced mortality within 14 days of the first positive blood culture. Definitive endocarditis was defined by Duke criteria (8). Patients were identified using Boolean search terms “endocarditis” and “Serratia,” “Klebsiella,” ”Escherichia,” *“E. coli*,” “Enterobacter,” “Citrobacter,” “Yersinia,” “Morganella,” “Providencia,” “Salmonella,” “Raoultella,” “Pantoea,” “Hafnia,” or “Proteus” as previously reported (7, 9).

Baseline ceftriaxone resistance was defined as the underlying pathogen harboring a ceftriaxone minimum inhibitory concentration of >1 µg/mL. Patients with polymicrobial blood cultures persistently growing gram-positive bacteria or yeast were not considered to have Enterobacterales endocarditis, unless the cultured valve grew the same Enterobacterales pathogen that was identified in blood cultures (9). In the setting of endocarditis caused by >1 Enterobacterales spp., the pathogen grown from valve cultures was considered the cause of endocarditis. If valve cultures were not available, or if valve cultures were also polymicrobial, the pathogen most commonly isolated from repeat blood cultures was used for analysis.

Combination therapy was defined as receipt of >1 antimicrobial agent with documented *in vitro* activity against the primary pathogen. Patients who received short-course combination treatment were compared to those who received long-course combination treatment. Short-course combination treatment was defined as receipt of combination therapy for ≤14 days, while long-course combination treatment was defined as receipt of combination therapy for >14 days. Indications for surgery included embolic prevention, uncontrolled infection, intracardiac complications, valve failure, or vegetation on an implantable cardiac device (9, 10). Combination regimens were classified as either a beta-lactam plus an aminoglycoside (AG), a beta-lactam plus a fluoroquinolone (FQ), or other combinations. Among patients who transitioned combination regimens due to an ADR or provider preference, the regimen that had been administered for the greatest number of days was used for analysis. Long-term failure was defined as a composite of 90-day mortality and 9-month recurrence.

Categorical variables were compared by a chi-squared or Fisher’s exact test. Continuous variables were compared using a Wilcoxon rank-sum test. A multivariate logistic regression analysis was performed to assess predictors of 90-day mortality and long-term failure. Factors with a *P*-value of <0.1 and no missing data from the univariate model were included in the multivariate model. Statistical significance was defined as a *P* value of < 0.05 (two-tailed). All analyses were performed using Stata, version 15.1 (College Station, TX, USA).

## RESULTS

There were 73 patients who met the inclusion criteria (Fig. S1). The median (IQR) age was 42 (32–65), and 63% (46/73) were people who inject drugs. The underlying pathogens were *S. marcescens* (62% [45/73]), *E. coli* (15% [11/73]), *K. oxytoca* (7% [5/73]), *K. pneumoniae* (7% [5/73]), *E. cloacae* complex (4% [3/73]), *P. mirabilis* (3% [2/73]), *S. liquefaciens* (1% [1/73]), and *K. aerogenes* (1% [1/73]). Polymicrobial infection occurred in 16% (12/73) of patients and was due to gram-positive (*n* = 6), gram-negative (*n* = 5), or *Candida* spp. (*n* = 1). Baseline ceftriaxone resistance occurred in 14% (10/73) of patients. Ceftriaxone resistance was observed in *S. marcescens* (*n* = 5), *E. coli* (*n* = 2), *E. cloacae* complex (*n* = 1), *K. aerogenes* (*n* = 1), and *K. pneumoniae* (*n* = 1).

Overall, 64% (47/73) of patients had a surgical indication for cardiac surgery. Surgical indications included embolic prevention alone (*n* = 11), valve failure alone (*n* = 7), an intracardiac complication alone (*n* = 1), an implantable cardiac electronic device in addition to valvular endocarditis (*n* = 1), and multiple indications (*n* = 27). Of the 27 patients with multiple indications, 17 had an intracardiac complication. Of the 18 patients with an intracardiac complication, 10 patients had a valve perforation, 6 had a valvular abscess, and 2 patients had both. Of the full cohort, 40% (29/73) of patients received surgery, and 25% (18/73) did not receive a surgical intervention despite an indication. Patients who did not receive a surgical intervention despite an indication had a higher Pitt bacteremia score (4 [1–5] vs 1 [0–3], *P* = 0.035) and a higher Charlson Comorbidity Index (4 [1–6] vs 1 [0–2], *P* = 0.005) compared to patients who either received surgery or did not have an indication. The median (IQR) time to surgical management from the time of the first positive blood culture was 12 days (6–22).

A beta-lactam was used as the primary treatment in 99% (72/73) of patients, and 1% (1/73) of patients received levofloxacin as the primary agent. Of the 72 patients who received a beta-lactam as the primary agent, treatments consisted of cefepime (*n* = 32), a third-generation cephalosporin (*n* = 22), a carbapenem (*n* = 12), piperacillin–tazobactam (*n* = 4), or a first-generation cephalosporin (*n* = 2). Combination regimens included a beta-lactam plus an AG (*n* = 30), a beta-lactam plus an FQ (*n* = 30), and other combinations (*n* = 13). Overall, 16% (12/73) of patients transitioned the combination regimen either due to an ADR (*n* = 8) or provider preference (*n* = 4).

Of the full cohort, 25% (18/73) of patients experienced an ADR requiring discontinuation of any antimicrobial agent, while 19% (14/73) experienced an ADR requiring discontinuation of the combination agent only. Of the 14 patients who experienced an ADR of the combination agent only, patients were transitioned to a different combination regimen (*n* = 8) or continued monotherapy (*n* = 6). ADRs included nephrotoxicity secondary to an AG (*n* = 6), QTc prolongation from a FQ (*n* = 4), ototoxicity from gentamicin (*n* = 1), neurotoxicity from cefepime (*n* = 2), transaminitis from ciprofloxacin (*n* = 1), transaminitis from cefazolin (*n* = 1), gastrointestinal intolerance from ceftriaxone (*n* = 1), or a rash from either cefepime or trimethoprim-sulfamethoxazole (*n* = 1). The final patient had experienced both neurotoxicity from cefepime and QTc prolongation from ciprofloxacin. The overall 90-day mortality rate and 9-month recurrence rate among 90-day survivors were 14% (10/73) and 11% (7/62), respectively. There was one patient who received short-course combination treatment in which 9-month outcomes were not yet available at the time of analysis. No patients experienced treatment-emergent resistance, and no cases of resistance were observed to either the primary or combination agent among patients with recurrence.

Of the included patients, 37% (27/73) of patients who received short-course combination treatment and 63% (46/73) received long-course combination treatment. The median (IQR) days of combination treatment for patients who received short-course and long-course combination treatment were 7 (4–10) and 42 (32–47), respectively. In the bivariate model, patients who received short-course combination treatment were significantly more likely to be admitted prior to 2016 (44% [12/27] vs 17% [8/46], *P* = 0.012), were significantly more likely to be treated with an AG combination (63% [17/27] vs 28% [13/46], *P* = 0.013), had a numerically higher Pitt Bacteremia score (3 [1–6] vs 1 [0–2], *P* = 0.075), and had a numerically shorter time to surgical management (11 [6–12] days vs 15 [10–23], *P* = 0.073) compared to those who received long-course combination treatment (Table 1). Despite these confounders, there was no significant difference in 90-day mortality (22% [6/27] vs 9% [4/46], *P* = 0.158), 90-day recurrence among 90-day survivors (15% [3/20] vs 10% [4/42], *P* = 0.671), or long-term failure (35% [9/26] vs 17% [8/46], *P* = 0.098). However, among 90-day survivors, patients who received short-course combination treatment had a shorter duration of post-infection length of stay compared to patients who received long-course combination treatment (18 [13–25] days vs 29 [20–42], *P* = 0.021). In a multivariate logistic regression model, short-course combination treatment was not associated with increased 90-day mortality (OR = 2.89; 95% CI: 0.63–13.35; *P* = 0.175) or long-term failure (OR = 1.72; 95% CI: 0.48–6.15; *P* = 0.402) (Table 2).

**TABLE 1:** Univariate comparison of short-course (≤14 days) or long-course (>14 days) combination treatment^*g*^

	Short course (*n* = 27)	Long course (*n* = 46)	Univariate *P*-value
Patient demographics and underlying diseases			
Age, median (IQR)	45 (31–74)	41 (34–58)	0.807
Female gender, *n* (%)	12 (44)	18 (39)	0.806
Admission prior to 2016, *n* (%)	12 (44)	8 (17)	**0.012**
Pitt bacteremia score, median (IQR)	3 (1–6)	1 (0–2)	0.075
Charlson comorbidity index, median (IQR)	1 (0–3)	1 (0–3)	0.995
People who inject drugs, *n* (%)	15 (56)	31 (67)	0.312
History of endocarditis, *n* (%)	9 (33)	19 (41)	0.499
Failed monotherapy for gram-negative endocarditis, *n* (%)	0 (0)	5 (11)	0.151
Infection characteristics			
Intracardiac complication, *n* (%)	7 (26)	11 (24)	0.847
Vegetation > 1 centimeter, *n* (%)	17 (63)	31 (67)	0.701
Prosthetic valve endocarditis, *n* (%)	7 (26)	9 (20)	0.567
Left-sided or multiple valve involvement, *n* (%)	17 (63)	35 (76)	0.232
Central nervous system septic emboli, *n* (%)	8 (30)	19 (41)	0.319
Septic emboli, *n* (%)	19 (70)	35 (76)	0.591
Concomitant vertebral osteomyelitis, *n* (%)	1 (4)	6 (13)	0.248
*Serratia spp*. as the underlying primary pathogen	16 (59)	30 (65)	0.611
Secondary endocarditis pathogen, *n* (%)	4 (15)	8 (17)	0.999
Gram-positive or *Candida* spp. polymicrobial infection, *n* (%)^*a*^	3 (11)	4 (9)	0.705
Ceftriaxone resistance, *n* (%)	2 (7)	8 (17)	0.305
Positive repeat blood cultures following active therapy, *n* (%)	11 (41)	16 (35)	0.611
Days to blood culture sterilization, median (IQR)	*N* = 11 3 (2–6)	*N* = 16 3 (2–4)	0.689
Treatment characteristics and source control			
Regimen *n* (%)			**0.013**
Beta-lactam + aminoglycoside	17 (63)	13 (28)	
Beta-lactam + fluoroquinolone	8 (30)	22 (48)	
Other combinations^*b*^	2 (7)	11 (24)	
Days to combination treatment, median (IQR)^*c*^	2 (2–5)	4 (1–7)	0.359
No surgical intervention despite an indication, *n* (%)	7 (26)	11 (24)	0.847
Days of antimicrobial treatment, median (IQR)	47 (42–53)	44 (42–54)	0.587
Days of combination treatment, median (IQR)	7 (4–10)	42 (32–47)	**<0.001**
Valve surgical management, *n* (%)	10 (37)	19 (41)	0.719
Days to surgical management, median (IQR)^*c*^	*N* = 10 11 (6–12)	*N* = 19 15 (10–23)	0.073
Positive valve culture, *n* (%)	*N* = 9 5 (56)	*N* = 15 5 (33)	0.403
Outcomes			
Any antimicrobial agent stopped early due to ADR, *n* (%)	7 (26)	11 (24)	0.847
Combination antimicrobial stopped early due to ADR, *n* (%)	7 (26)	7 (15)	0.262
42-day mortality, *n* (%)	5 (19)	3 (7)	0.137
90-day mortality, *n* (%)	6 (22)	4 (9)	0.158
Post-infection length of stay, median (IQR) (3)	*N* = 21 18 (13–25)	*N* = 42 29 (20–42)	**0.021**
9-month recurrence, *n* (%)^*d*^^,^^*e*^^,^^*f*^	*N* = 20 3 (15)	*N* = 42 4 (10)	0.671
Long-term failure, *n* (%)^*e*^	*N* = 26 9 (35)	*N* = 46 8 (17)	0.098

^*a*^ Underlying pathogens were due to *Staphylococcus aureus* (*n *= 2), Viridans Group *Streptococci* (*n *= 2), *Enterococcus faecalis* (*n *= 1), *Micrococcus *spp. (*n *= 1), and *Candida *spp. (*n *= 1). There were no patients with a polymicrobial infection due to Gram-positive bacteria or Candida spp. who experienced 90-day mortality.

^*b*^ Other combinations consisted of a beta-lactam plus trimethoprim-sulfamethoxazole (*n *= 8), meropenem plus colistin (*n *= 1), levofloxacin plus colistin (*n *= 1), a beta-lactam plus doxycycline (*n *= 2), and ceftriaxone plus ampicillin (*n *= 1).

^*c*^ From the time of the first positive blood culture.

^*d*^ Among 90-day survivors.

^*e*^ 9 months has not passed yet for one patient.

^*f*^ No cases of resistance were seen to either the primary or backbone agent among patients with recurrence (*n* = 7).

^*g*^ ADR, adverse drug reaction; *P*-values bolded if < 0.05.

**TABLE 2:** Multivariate logistic regression model^*c*^

90-day mortality^*a*^			
	OR	95% CI	*P*-value
Admission prior to 2016	0.75	0.14–4.09	0.739
Pitt bacteremia score	1.04	0.82–1.31	0.765
Beta-lactam +aminoglycoside	1.18	0.24–5.81	0.831
Short course	2.89	0.63–13.35	0.175

Long-term failure^*b*^			
Admission prior to 2016	1.07	0.26–4.42	0.924
Pitt bacteremia score	1.19	0.98–1.46	0.076
Beta-lactam +aminoglycoside	1.41	0.36–5.46	0.621
Short course	1.72	0.48–6.15	0.402

^*a*^ Hosmer-Lemeshow goodness-of-fit test: X^2 ^= 29.7; *P *= 0.48.

^*b*^ Hosmer-Lemeshow goodness-of-fit test: X^2 ^= 36.7; *P *= 0.155.

^*c*^ In the bivariate model, patients who received short-course treatment had a numerically shorter time to surgical management (11 [6–12] days vs 15 [10–23], *P *= 0.073); However, this factor was excluded from the model due to sample size.

## DISCUSSION

This is the first study to evaluate the duration of combination therapy in patients with Enterobacterales endocarditis. It is notable that treatment-emergent resistance was not observed in this study of patients who received combination therapy but was observed among those who had received monotherapy for endocarditis secondary to *Serratia* and other Enterobacterales in our prior work (1, 7). Given the absence of treatment-emergent resistance in either group within the present study, these data would support short-course combination therapy. Although not significantly different, 90-day mortality rates were numerically higher in the short-course combination group. However, it is hypothesized that this numeric difference is attributed to a higher baseline Pitt bacteremia score, considering this variable has been independently associated with failure for patients with gram-negative endocarditis (9). Notably, the 90-day mortality rate among patients who received short-course combination therapy (22% [6/27]) remains lower than our previously reported 90-day mortality rates among patients who received monotherapy for *Serratia* endocarditis (31% [12/39]) and valvular endocarditis due to other Enterobacterales (37% [16/43]) (1, 7).

While combination therapy has been associated with decreased mortality in the treatment of Enterobacterales endocarditis compared to monotherapy, it has also been shown to have an increased risk of ADRs compared to monotherapy (1, 5, 7). Therefore, shorter durations of combination antimicrobial exposure may mitigate these adverse effects. Indeed, shorter courses of combination therapy prior to narrowing to monotherapy have been shown to be as efficacious for *S. aureus* and *E. faecalis* endocarditis compared to a full 6-week course of combination therapy (11, 12). Consistent with the present study, use of short-course combination therapy in *E. faecalis* endocarditis has been associated with a decreased length of hospitalization (11).

The largest study from the United States indicates that the majority of patients receive monotherapy for non-HACEK gram-negative endocarditis, whereas the largest European study found combination therapy to be more commonly administered (9, 13). Notable data from this real-world study are that short-course combination therapy was less commonly prescribed compared to long-course combination treatment, which may be reflective of current guideline recommendations to administer 6 weeks of combination treatment (8). Patients in the short-course group were more likely to receive an AG, and it may be postulated that combination treatment was discontinued early since the majority of ADRs occurred due to an AG. It is also recognized that there may have been a change in clinical practice in either the duration of combination treatment or combination regimen used since patients who received short-course treatment were more likely to be admitted prior to 2016. This shift in practice to avoid AG for gram-negative endocarditis may have been influenced by the GAMES investigators, who reported improved mortality rates when a FQ was used as part of the combination regimen (13). Although the multivariate model in the present study did not indicate an association of AG use with mortality, our definition of the combination regimen was based on days of therapy instead of the initial regimen used. The purpose of the present study was not to identify the optimal agent to be used in combination for Enterobacterales endocarditis; however, this represents an area for further research.

While the strength of this study lies in its ability to better elucidate the treatment of a rare but life-threatening disease, several limitations in this study are recognized. First, this was an observational study, and the small sample size leaves this study vulnerable to a type II statistical error. However, given that this data were aggregated across 11 centers over a quarter of a century, larger studies may not be possible. Despite meeting criteria for definitive endocarditis due to Enterobacterales, over half of the patients received exclusive monotherapy, which limited our sample size. We also excluded patients with endocarditis due to *P. aeruginosa* since no clinical studies to date have identified an association between combination therapy and improved outcomes; however, further research is still required in this area (5, 6). Second, the occurrence of an ADR frequently influenced the decision to terminate combination therapy. In turn, this hindered our ability to detect a significant difference in the rate of ADRs between short-course and long-course groups. Third, several baseline confounders are present in the univariate model, suggesting a higher severity of illness in the short-course combination group. However, after applying multivariate logistic regression, short-course combination treatment was not associated with worse outcomes. Finally, the decision to define short-course combination treatment as receipt of therapy for ≤14 days was inspired by a randomized trial evaluating 2 weeks of combination therapy for the treatment of endocarditis caused by Viridans group Streptococci (14). Given the limited data to guide the duration of treatment for gram-negative endocarditis, future studies with larger sample sizes should focus on more nuanced durations. For instance, data from MRSA endocarditis would support using monotherapy following blood culture sterilization and adequate source control (6). Despite these limitations, shorter courses of combination therapy were commonly prescribed, which supports future studies that are designed to identify the optimal duration of combination therapy for patients with Enterobacterales endocarditis.

## SUPPLEMENTAL MATERIAL

Fig. S1 aac.00357-26-s0001.png Inclusion and exclusion criteria.

ASM does not own the copyrights to Supplemental Material that may be linked to, or accessed through, an article. The authors have granted ASM a non-exclusive, world-wide license to publish the Supplemental Material files. Please contact the corresponding author directly for reuse.
