# Supplementary figures and images for: Evaluation of combination therapy duration for endocarditis secondary to Enterobacterales
Source: Antimicrob Agents Chemother. 2026 May 28;70(7):e00357-26. doi: 10.1128/aac.00357-26 (PMC13321831; doi:10.1128/aac.00357-26)

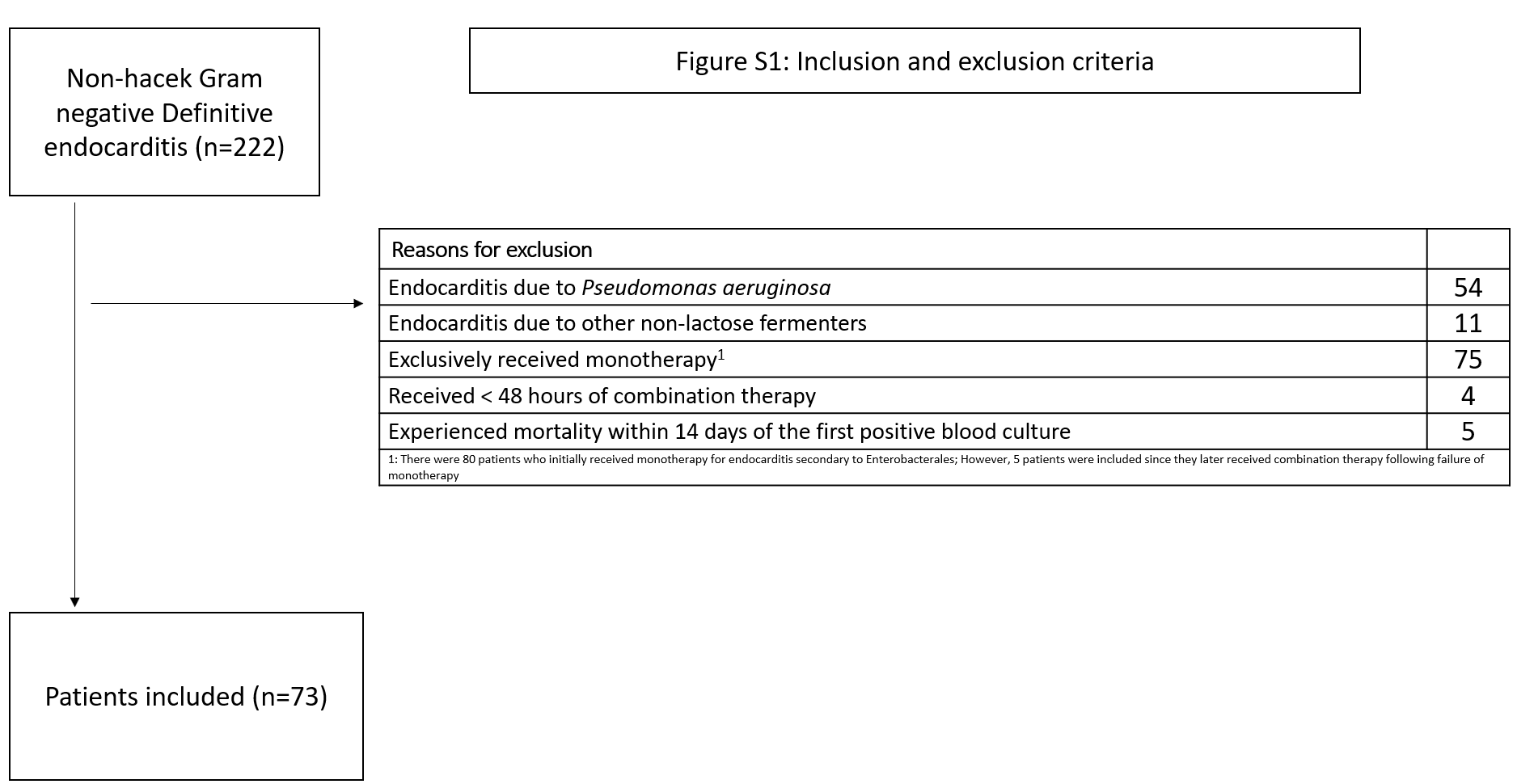

Supplement: Fig. S1 — Inclusion and exclusion criteria. [file aac.00357-26-s0001.png]
